# Assessment of Genetic Diversity among Barley Cultivars and Breeding Lines Adapted to the US Pacific Northwest, and Its Implications in Breeding Barley for Imidazolinone-Resistance

**DOI:** 10.1371/journal.pone.0100998

**Published:** 2014-06-26

**Authors:** Sachin Rustgi, Janet Matanguihan, Jaime H. Mejías, Richa Gemini, Rhoda A. T. Brew-Appiah, Nuan Wen, Claudia Osorio, Nii Ankrah, Kevin M. Murphy, Diter von Wettstein

**Affiliations:** 1 Department of Crop & Soil Sciences, Washington State University, Pullman, Washington, United States of America; 2 Instituto de Investigaciones Agropecuarias INIA, Vilcún, Chile; 3 School of Molecular Biosciences, Washington State University, Pullman, Washington, United States of America; 4 Centre for Reproductive Biology, Washington State University, Pullman, Washington, United States of America; Nanjing Agricultural University, China

## Abstract

Extensive application of imidazolinone (IMI) herbicides had a significant impact on barley productivity contributing to a continuous decline in its acreage over the last two decades. A possible solution to this problem is to transfer IMI-resistance from a recently characterized mutation in the ‘Bob’ barley *AHAS* (*acetohydroxy acid synthase*) gene to other food, feed and malting barley cultivars. We focused our efforts on transferring IMI-resistance to barley varieties adapted to the US Pacific Northwest (PNW), since it comprises ∼23% (335,000 ha) of the US agricultural land under barley production. To effectively breed for IMI-resistance, we studied the genetic diversity among 13 two-rowed spring barley cultivars/breeding-lines from the PNW using 61 microsatellite markers, and selected six barley genotypes that showed medium to high genetic dissimilarity with the ‘Bob’ *AHAS* mutant. The six selected genotypes were used to make 29–53 crosses with the *AHAS* mutant and a range of 358–471 F_1_ seeds were obtained. To make informed selection for the recovery of the recipient parent genome, the genetic location of the *AHAS* gene was determined and its genetic nature assessed. Large F_2_ populations ranging in size from 2158–2846 individuals were evaluated for herbicide resistance and seedling vigor. Based on the results, F_3_ lines from the six most vigorous F_2_ genotypes per cross combination were evaluated for their genetic background. A range of 20%–90% recovery of the recipient parent genome for the carrier chromosome was observed. An effort was made to determine the critical dose of herbicide to distinguish between heterozygotes and homozygotes for the mutant allele. Results suggested that the mutant can survive up to the 10× field recommended dose of herbicide, and the 8× and 10× herbicide doses can distinguish between the two *AHAS* mutant genotypes. Finally, implications of this research in sustaining barley productivity in the PNW are discussed.

## Introduction

Barley is a short-season, early maturing annual grain crop with some degree of tolerance to drought and salinity, which allows its production in a wide range of climatic zones including both irrigated and dryland production areas [1]. Barley is the third major feed grain crop produced in the United States, after corn and sorghum [2]. Spring barley is a preferred rotational crop in the US Pacific Northwest (PNW) for two- or three-year rotations with winter wheat (*Triticum aestivum* L.), pea (*Pisum sativum* L.), lentil (*Lens culinaris* L.), or fallow [1], [3]. A cropping system like spring wheat-fallow or winter wheat-fallow is generally practiced in the PNW, which encourages populations of summer and winter annual-grassy weeds, respectively [4]. These weed cycles can be broken with a winter wheat-barley-fallow rotation [6]. Depending upon the management practices followed in an area, this cropping system results in a buildup of crown and root rot pathogens including *Fusarium*, *Rhizoctonia* and *Phythium* species, which frequently result in significant yield losses [5]. Similarly, in an eight-year dryland no-till cropping systems experiment conducted near Ritzville, Washington, a significant drop in the incidence of bare patches caused by *Rhizoctonia* was observed by adaptation of a two-year spring wheat rotation with spring barley. A significant gain in average yield of spring wheat was also documented with this change [5]. Likewise, in continuous cropping systems, spring barley fits well after winter wheat because the time interval between harvesting the barley crop and planting winter wheat is usually sufficient to allow soil moisture recharge to support an optimum winter wheat stand [6], [7]. In addition to its agronomical relevance and commercial value as a feed or malt grain crop, barley is regaining popularity as human food due to the antioxidant and β-glucan (dietary fiber) rich grains [8], [9]. Despite its agronomical importance and rising market value, barley acreage in the US has declined from 8.94 million acres in 1991 to 3.48 million acres in 2013 [10]. In Washington State alone the acreage has dropped significantly from 500,000 acres planted in 1999 to 180,000 acres in 2013 [10].

The significant drop in barley acreage during the last two decades can be partly attributed to the wide scale application of imidazolinone herbicides in combination with the introduction of imidazolinone (IMI)-resistant crops, and the residual activity of the herbicides of this family [1]. The decline in acreage can also be explained by the overlapping distribution of regions under barley cultivation in the PNW and the regions under extensive application of Imazamox (Beyond) and/or Imazethapyr (Pursuit) [11]. Collectively, the major reason for the decline in barley acreage is its sensitivity to commonly used herbicides. Many of the widely used herbicides, which impose barley plant-back restrictions, belong to the group B herbicides [12]. Thus, identification of IMI-resistant mutant(s) in barley and its transfer to relevant feed, food and/or malting barley cultivars adapted to the PNW is of extreme importance to sustain barley productivity in this region and elsewhere.

The group of herbicides belonging to the imidazolinone family targets acetohydroxyacid synthase (AHAS) or acetolactate synthase (ALS), an octameric enzyme with four catalytic and four regulatory subunits [13]. The enzyme AHAS catalyses two parallel reactions in the synthesis of branched chain amino acids. The first reaction is condensation of two pyruvate molecules to yield acetolactate leading to the production of valine and leucine, and the other reaction is the condensation of pyruvate and *α*-ketobutyrate that give rise to acetohydroxybutyrate, which subsequently results in the synthesis of isoleucine [14]. The AHAS-inhibiting herbicides are known to bind at the substrate access channel, blocking the path of substrate to the active site. When AHAS is inhibited, deficiency of the amino acids (valine, leucine and isoleucine) causes a decrease in protein synthesis, which in turn slows down the rate of cell division. This process eventually kills the plant, with symptoms observed in meristematic tissues where biosynthesis of amino acids primarily takes place [12]. In most cases, resistant plants have a reduced sensitivity to these herbicides due to amino acid substitution(s) in AHAS that give rise to catalytically active isoforms of the enzyme. Most AHAS isoenzymes resistant to the herbicides carry substitutions for the amino acid residues Ala122, Pro197, Ala205, Asp376, Trp574 or Ser653 (amino acid numbering refers to the sequence in *Arabidopsis thaliana*) [13]. Amino acid substitutions at Ala122 and Ser653 confer high levels of resistance to imidazolinone herbicides, whereas substitutions at Pro197 endow high level of resistance against sulfonylureas and provide low-level resistance against imidazolinone and triazolopyrimidine herbicides. Likewise, substitutions at Trp574 provide high levels of resistance to imidazolinones, sulfonylureas and triazolopyrimidines, while substitutions at Ala205 confer resistance against all AHAS-inhibiting herbicides [15].

In the case of barley, there is no IMI-resistance reported for any of the varieties cultivated in the PNW. Thus, introduction of a barley variety with IMI-resistance will provide greater flexibility to barley as a rotational crop after winter wheat [11]. An IMI-resistant mutant was earlier isolated by our group from an extensive screening of two million seeds of ‘Bob’ treated with sodium azide. Molecular characterization of the mutant revealed an amino acid substitution in the substrate access channel of the catalytic subunit of the AHAS enzyme, changing a serine to asparagine at amino acid location 653 [16]. This mutation in the substrate access channel does not allow imazamox to block the path of the substrate to the active site, thus allowing the plant to survive with no obvious effects on plant fitness even when exposed to field recommended dose of herbicide used on the IMI-tolerant winter wheat (i.e., 0.118 L/Acre Beyond with 1% non-ionic surfactant).

In view of the agronomical importance of this trait and the great demand for IMI-resistant barley cultivars in the PNW, this study was undertaken with the following objectives: i) estimation of genetic diversity among the 13 two-rowed spring barley cultivars/breeding-lines adapted to the US PNW using 61 microsatellite markers to select for lines showing sufficient genotypic differences with the ‘Bob’ *AHAS* mutant, to be used in the crossing program; and (ii) transfer the IMI-resistance to selected food, feed and malting barley cultivars using marker-assisted foreground and background selections.

## Materials and Methods

### Plant material

Seeds of the 13 two-rowed spring barley cultivars or breeding lines were procured from the variety testing program at the Washington State University (WSU), Pullman. Of the 13 genotypes selected for genetic analysis, eight are feed barleys, three are food barleys and the remaining two are malting barleys (Table 1).

**Table 1:** List of two-rowed spring barley varieties/breeding lines used in the study.

Genotype	Pedigree	Class
Baronesse	([(Mentor×Minerva)×mutant ofVada]×[(Carlsberg×Union)×(Opavsky×Salle)×Ricardo])×(Oriol×6153 P40)	hulled, feed barley (originally released as malting barley)
Bob	(Lewis somaclonal line)/Baronesse	hulled, feed barley
Champion	Baronesse/Camas	hulled, feed barley
Clearwater	Baronesse*2/pmut882//HB317(CDC Dawn sib)	hulless, low phytate, food barley
Lenetah	94Ab12981/Criton	hulled, feed barley
Conrad	B1215/B88–5336	hulled, malting barley
Radiant	*ant29–667* (an induced mutant in Harrington)/Baronesse	hulled, malting barley, pro-anthocyanidine-free
Spaulding	Vanguard/Imber//Zephyr/3/Heavyweight/4/VD403582	hulled, feed barley
WAS4	01WA-13862.3/Radiant	hulless, food barley
05WA-316.99	Baronesse/Spaulding	hulled, feed barley
Lyon	Baronesse/Spaulding	hulled, feed barley
07WA-682.1	WA 10701–99/AC Metcalfe	hulled, feed barley
Meresse	Merlin/Baronesse	hulless, food barley

### Crossing scheme

To transfer IMI-resistance from the ‘Bob’ *AHAS* mutant, crosses were made between the mutant and each of the six barley genotypes, selected on the basis of genetic diversity analysis performed using microsatellite markers specific to chromosome 6H (see later for details). Twenty nine to fifty three crosses were made per genotype combination during the summers of 2012 at the Spillman Agronomy Farm (WSU, Pullman) and a range of 358 to 471 F_1_ grains were harvested. The F_1_ plants were propagated in 48-well flats in the glasshouse to obtain F_2_ seeds. Subsequently, a range 2158 to 2846 F_2_ plants per cross combination were evaluated for herbicide resistance by spraying two-week-old seedlings with 0.236 L/Acer Beyond (twice the field recommended dose applied to the IMI-tolerant winter wheat) with 1% methylated seed oil (MSO). A month after herbicide spray, the survivors (i.e., resistant plants) were evaluated for plant height as an indicator of early vigor and the 250 top ranking lines per cross combination were raised to maturity for seeds. Later, one to three F_3_ plants each from the six most vigorous F_2_ lines per cross combination were evaluated for the genotype at the *AHAS* locus by DNA sequencing, and the percent recovery of the recipient parent genome using chromosome 6H-specific SSR markers.

### DNA extraction and PCR amplification

DNA was extracted from the one-month-old seedlings of each of the 13 barley genotypes, and the two-week-old seedlings of the F_3_ progeny of selected F_2_ lines, using the modified CTAB (Cetyl Trimethyl Ammonium Bromide) method [17]. DNA was treated by RNAse and purified by phenol extraction (25 phenol: 24 chloroform: 1 isoamyl alcohol, v/v/v) followed by ethanol precipitation [18]. Concentration of DNA samples was adjusted to 50 ng µl^−1^ using *Hind* III digested λ DNA as a marker. DNA amplification was carried out on a C1000 thermal cycler (Bio-Rad Laboratories). The PCR reactions were performed in 20 µl reaction mixtures, each containing 50 ng template DNA, 0.25 µM primers, 200 µM dNTPs, 1.5 mM MgCl_2_, 1×PCR buffer and 0.5 U Ex *Taq* DNA polymerase (TAKARA, Bio Inc.) using the following PCR profile: initial denaturation at 95°C for 3 min followed by 40 cycles at 95°C for 30 sec, 53–61°C (depending upon the primer pair used) for 30 sec (for primer details, cf. [19]), 72°C for 45 sec, and a final extension at 72°C for 5 min. The amplification products were resolved on 10% polyacrylamide denaturing gels followed by silver staining [20]. A hundred base pair ladder was used as a size marker (New England BioLabs, Inc., Beverly, USA). The amplified product/allele sizes were determined using Fragment Size Calculator available at http://www.basic.northwestern.edu/biotools/SizeCalc.html.

### DNA sequencing and sequence analysis

To determine the genotype at the *AHAS* locus, genomic DNA extracted from the F_3_ progeny of selected F_2_ lines was amplified using the *AHAS* gene-specific sequence tagged site (STS) primers that flank the point mutation responsible for the IMI-resistance (for primer details, cf. [16]). The amplification product was resolved on 1% agarose gel. A 100-bp ladder was used as a size marker (New England BioLabs). The band of expected size was excised from the gel, and DNA was eluted from the band using the Geneclean kit following the manufacturer’s instructions (MP Biomedicals). The eluted DNA was used as a template for the sequencing reaction using either forward or reverse primers in separate reactions. The sequencing reactions were carried out at the DNA Sequence Core, WSU, Pullman. Alignment of the DNA sequences was performed using the Vector NTI AdvanceTM 9.1 (Invitrogen).

### Determination of the polymorphic information content (PIC) and genetic diversity

For each microsatellite or simple sequence repeat (SSR) locus, PIC was calculated using the following equation: PIC = 1–Σ(Pi)^2^, where Pi is the proportion of genotypes carrying the i^th^ allele [21]. For dissimilarity analysis, null alleles were scored as zero (0) and other microsatellite alleles (length variants) were each scored in the form of single bands of expected sizes, which were later converted into the number of repeat units as allele codes (all modalities were given equal weight during the analysis). The numerical data thus obtained was used to calculate Sokal and Michener dissimilarity indices (d*i*–*j*) [22]. The dissimilarity indices between pairs of accessions using genotypic data were calculated on the basis of the following equation: d*i*–*j* =  (n11+n00)/(n11+n01+n10+n00), where n11 is the number of fragments present in both *i* and *j*, n01 and n10 is the number of fragments present in one accession but absent in the other, and n00 is the number of fragments absent in both *i* and *j*. From the obtained distance matrix, an un-weighted Neighbor-Joining tree [23] was computed using the Darwin 5.0 software [24] and branch robustness was tested using 1000 bootstraps.

### Enzyme extraction

Soluble proteins from ‘Bob’ and ‘Bob’ *AHAS* mutant were extracted following Singh et al. [25], with minor modifications. Briefly, two batches of 500 mg of the fresh leaf tissue were pulverized each with 5 mL of the protein extraction buffer [consisting of 100 mM potassium phosphate buffer (pH 7.5), 10 mM sodium pyruvate, 5 mM MgCl_2_, 5 mM EDTA, 100 µM flavin adenine dinucleotide (FAD) and 10% Glycerol], using a polypropylene mesh bag (supplied with the P-PER Plant Protein Extraction Kit, Thermo Scientific). After adding the extraction buffer to the leaf tissue, the bag was rubbed from the outside with a ceramic pestle until a homogeneous mixture of the tissue was obtained. Later, the lysate was suctioned from the bag using a pipette and placed into a 15 mL conical tube and centrifuged at 22,000×g for 20 min at 4**°**C. The supernatant was transferred to a new tube and mixed with an equal volume of saturated (NH_4_)_2_SO_4_. The mixture was incubated on ice for 30 min, and then centrifuged at 4**°**C for 20 min at 22,000×g. The supernatant was discarded and the pellet containing protein was re-suspended in 700 µL of the buffer solution containing 50 mM potassium phosphate (pH 7.5), 100 mM sodium pyruvate, 10 mM MgCl_2_, 1 mM EDTA, 10 µM FAD, 100 mM NaCl and 1 mM thiamine pyrophosphate (TPP).

After extraction, protein concentration was determined using Bradford colorimetric micro-assay by mixing 80 µL of protein extract with 20 µL of the Bradford reagent (containing 1 mL of concentrated Bradford solution in 4 mL of deionized water), and measuring absorbance at 590 nm wavelength. The presence of the enzyme in the extract was also confirmed by loading protein extracts on 10% sodium dodecyl sulfate (SDS) polyacrylamide gel. For this purpose 15 µL of protein extract was mixed with 3 µL of the loading buffer, and electrophoresed on polyacrylamide gel for 2 h at 120 volts. After electrophoresis, the gel was stained with Coomassie brilliant blue reagent (80% Coomassie and 20% methanol, v/v) for 24 h. A protein band of ∼65 kDa was observed, which corresponds with the size of AHAS enzyme monomers, confirming its presence in the extract.

### Colorimetric enzyme activity assay

Enzyme activity was tested by using five different doses of Beyond (i.e., 1×, 4×, 6×, 8× and 10× the field recommended dose applied on IMI-tolerant winter wheat) with 0.25% (v/v) nonionic surfactant (NIS). Initial reaction was performed in 1.5 mL microfuge tube by adding 52 µL of enzyme (in extraction buffer containing the substrate and co-factors, see above for the buffer composition) to equal volume of herbicide and incubating the mixture at 37**°**C for 1 h to facilitate acetolactate production. Later, the reaction was stopped by adding 21 µL of 5% H_2_SO_4_, and incubating at 60**°**C for 15 min. After incubation, tubes were spiked with 175 µL of color change solution containing 0.32 g of NaOH, 0.12 g of 1-naphtol and 0.01 g of creatine in 4 mL of deionized water, and the mixture was re-incubated at 60**°**C for 15 min. After incubation, 200 µL sub-samples of the reaction mixture were added to a 96-well microtiter plate (Falcon cat#353077) to determine the enzyme activity by studying color change using a microplate reader spectrophotometer (Spectra Max, M2, Molecular Devices) at 520 nm wavelength.

## Results and Discussion

### Chromosomal assignment of the gene encoding catalytic subunit of barley AHAS enzyme

The AHAS holoenzyme (∼548 kDa) consists of two halves where one half, known as the large or catalytic subunit, is comprised of a homotetramer of ∼65 kDa polypeptides, and the second half, known as the small or regulatory subunit, consists of homo-tetramer/-pentamer of polypeptides of ∼52 kDa each [16], [26], [27]. The regulatory subunit stimulates enzyme activity and is required for the feedback regulation of the branched-chain amino acid biosynthesis, whereas the catalytic subunit is solely responsible for the enzyme activity and is also the site of point mutation(s) that confers resistance against IMI-herbicides [16]. Due to the importance of the catalytic subunit in providing IMI-resistance, the genes encoding it have been studied in common wheat and assigned to group 6 chromosomes [6A (*imi3*), 6B (*imi2*) and 6D (*imi1*)], using nulli-tetrasomic lines [28]. Later, the genetic location of *imi1* gene on the long arm of chromosome 6D was determined using three mapping populations, namely Cashup/cv. 9804, Madsen/cv. 9804 and Opata 85/W7984 [28]. However, the genetic location of the *AHAS* gene in barley remains unknown. Therefore, we used the map location of the *AHAS* gene in wheat to decipher its location in barley, which is possible in this particular case due to the shared ancestry of the two genera, and high levels of synteny as well as colinearity between them [29]. The availability of common markers between wheat and barley maps allowed an approximation of the barley *AHAS* gene location on chromosome 6H (Fig. S1). Moreover, we used the complete *AHAS* gene sequence we had previously obtained to blast against the barley genomic DNA sequences available in the public domain (http://webblast.ipk-gatersleben.de/barley/). The BLASTn search (score = 2834 and E-value = 0.0) allowed unambiguous assignment of the gene to genetically anchored ‘Morex’ BAC contig numbered 40275 on chromosome 6H at 67.917 cM (Fig. S1). In addition, the initial genotyping of the F_3_ progeny of selected F_2_ lines (carrying the *AHAS* mutant allele in hetero-/homozygous state) from all six cross combinations with chromosome 6H specific microsatellite markers showed higher recovery rate (50–72%) of recipient parent alleles for markers mapping to the non-proximal long arm in comparison with the short arm and the centromeric region (37–58%) (see next section for details). This is an indication of suppressed recombination, likely due to selection for the trait of interest. Collectively, the *in silico* and experimental data strongly indicate that the gene encoding the catalytic subunit of the AHAS enzyme maps to the sub-centromeric region of the barley chromosome arm 6HL.

### Polymorphism survey using chromosome 6H-specific microsatellite markers

The level of genetic diversity among 13 two-rowed spring barley cultivars/breeding lines adapted to the PNW was assessed using microsatellite or simple-sequence repeat (SSR) markers specific to the barley chromosome 6H. Out of the 13 genotypes selected for the analysis, eight are feed barleys, three are food barleys and two are malting barleys (Table 1). The 61 SSR markers selected for the analysis are evenly distributed along the entire length of chromosome 6H (Table 2) [19]. The major reason behind selecting markers from chromosome 6H lies in the fact that this chromosome carries the gene encoding for the catalytic subunit of acetohydroxyacid synthase (AHAS) enzyme and the mutation providing IMI-resistance (see above). It is known through trait-introgression studies that due to linkage-drag, it always takes longer (several backcrossing and selfing generations) to recover the recipient parent genotype for the carrier chromosome in comparison with non-carrier chromosomes, which assort independently [30]. Thus, to identify the rare recombinant(s) carrying the precise gene introgression in the early generation, it is important to screen large segregating populations with the markers derived from the carrier chromosome.

**Table 2:** List of chromosome 6H specific microsatellite markers used for the genetic diversity analysis and marker-assisted background selection, their repeat elements, respective locations in the genetic-linkage map [17], number of alleles detected and their polymorphic information content (PIC).

Marker/loci	Repeat element	Position (cM)	PIC	Allele#
*Af166121*	(A)_10_	0.00	0.38	3
*84c21j33*	(T)_10_	0.00	0.14	2
*Bmac0316*	(AC)_19_	7.16	0.80	7
*scssr09398*	(CTT)_9_	7.16	0.43	2
*Bmag0500*	(AG)_6_CG(AG)_29_(AGAGGG)_3_(AG)_6_	31.65	0.72	6
*GBM1270*	(GCC)_8_	36.52	0.56	4
*GBM1355*	(GCA)_7_	40.43	0.14	2
*GBM1212*	(AGG)_5_	55.10	0.14	2
*Bmag0807*	(TC)_18_	56.11	0.39	4
*Bmag0173*	(CT)_29_	57.79	0.86	9
*GBM1423*	(CGGCTC)_5_	58.46	0.36	2
*HVM31*	(AC)_9_	60.90	0.57	3
*Bmac0040*	(AC)_20_	61.07	0.77	7
*Bmag0174*	(AG)_9_	61.40	0.72	6
*EBmac0560*	(AC)_7_	61.70	0.77	5
*GBM1267*	(TTG)_9_	61.70	0.69	4
*Bmac0018*	(AC)_11_	61.79	0.77	7
*Bmac0144*	(AT)_4_(AC)_20_	61.79	0.91	12
*Bmac0175*	(AC)_12_	61.79	0.57	3
*GBM5012*	(ACG)_7_	61.95	0.49	2
*Ebmac0674*	(TG)_18_(AG)_9_	61.96	0.46	3
*EBmac0874.1*	(CA)_8_AA(CA)_4_CG(CA)_8_AA(CA)_7_AA(CA)_9_(TA)_8_	61.96	0.67	4
*EBmac0874.2*	(CA)_8_AA(CA)_4_CG(CA)_8_AA(CA)_7_AA(CA)_9_(TA)_8_	61.96	0.80	7
*HVM65*	(GA)_10_	62.11	0.71	6
*Bmag0009*	(AG)_13_	62.21	0.57	3
*Ebmac0639*	(TG)_5_(TG)_8_	62.21	0.57	4
*EBmatc0028.1*	(ATC)_3_N3(ATC)_6_	62.21	0.50	2
*EBmatc0028.2*	(ATC)_3_N3(ATC)_6_	62.21	0.58	4
*EBmatc0028.3*	(ATC)_3_N3(ATC)_6_	62.21	0.67	5
*Bmac0297.1*	(AC)_9_(AC)_10_	62.23	0.58	4
*Bmac0297.2*	(AC)_9_(AC)_10_	62.23	0.77	5
*Bmac0297.3*	(AC)_9_(AC)_10_	62.23	0.46	3
*Bmac0047*	(AC)_16_	62.27	0.47	2
*Bmac0127*	(AC)_26_	62.27	0.47	2
*GBM1389*	(GCCT)_5_	62.27	0.26	2
*HVM14*	(CA)_11_	62.28	0.50	2
*Bmag210*	(AG)_7_T(AG)_13_	62.28	0.57	3
*HVM34*	(GA)_10_	62.43	0.36	2
*HVM74*	(GA)_13_	62.66	0.56	3
*Bmag0003.1*	(AG)_28_	63.49	0.78	6
*Bmag0003.2*	(AG)_28_	63.49	0.59	6
*Bmag0004*	(AG)_14_	64.71	0.91	12
*BMG001*	(G)_10_	64.71	0.52	3
*Bmgt0001*	(GTTTTT)_5_	64.71	0.27	3
*scssr02093*	(GA)_18_	67.20	0.49	2
*Bmag0344*	(CT)_10_GT(CT)_16_	67.80	0.66	6
*GBM1400*	(CACG)_5_	67.80	0.27	3
*Bmac0251*	(AC)_12_A(AC)_13_	69.25	0.38	3
*Bmag0613*	(GA)_17_	69.82	0.66	6
*Bmac0218*	(AC)_14_	71.99	0.79	6
*Bmac602*	(AC)_9_AT(AC)_7_(AG)_9_	75.42	0.49	4
*GBM1256*	(GA)_8_	75.46	0.52	3
*HVM11*	(GGA)_3_(GGA)(GAA)_2_	88.47	0.56	3
*scssr05599*	(AAG)_4_	96.34	0.63	4
*GBM1140*	(ATC)_5_	97.31	0.52	3
*GBM1356*	(GTG)_7_	98.38	0.57	3
*scssr00103*	(GT)_10_	105.26	0.59	3
*GBM1274*	(TCG)_7_	123.45	0.27	3
*GBM1275*	(TGC)_7_	124.29	0.15	2
*GBM1276*	(TGC)_7_	124.29	0.46	3
*GBM1087*	(AGG)_5_	127.70	0.54	3
*GBM1404*	(TATG)_5_	129.76	0.46	3

**Figure 1:**
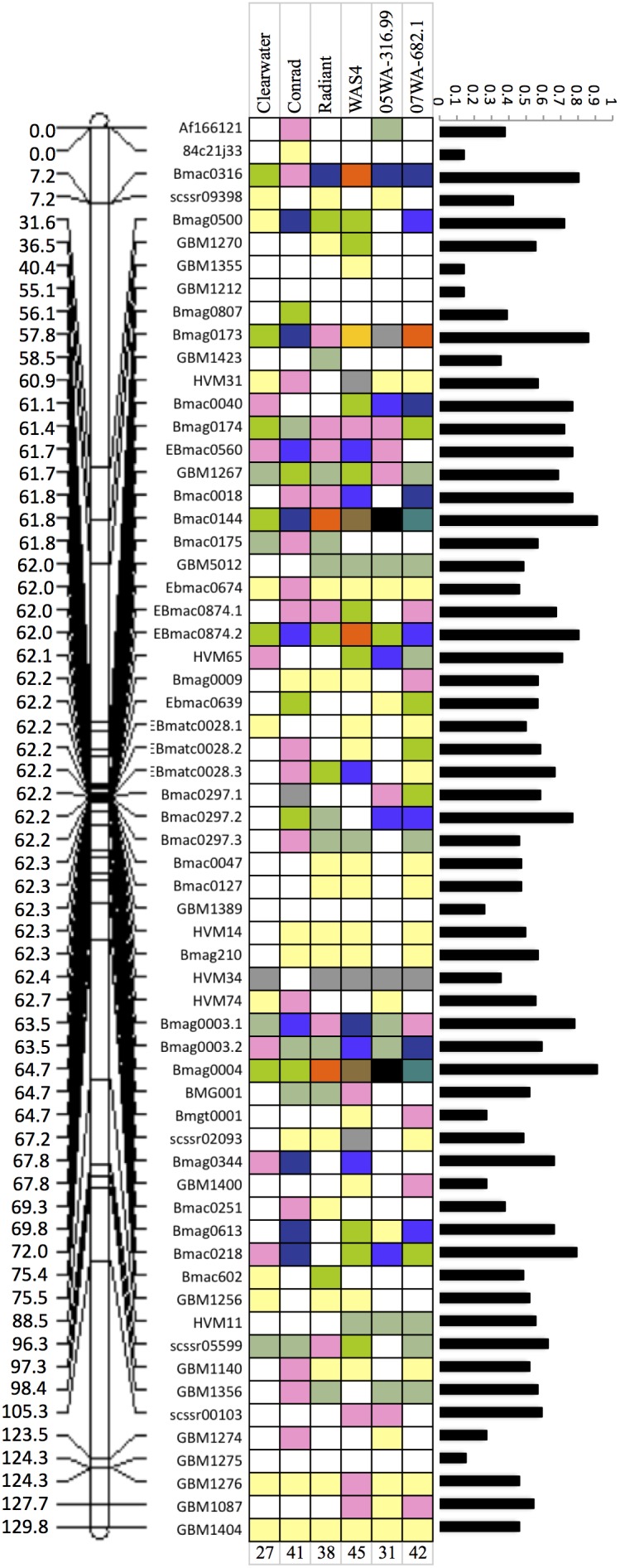
Genetic linkage map of chromosome 6H showing the respective locations of 61 microsatellite markers used in the present study (left). Various alleles detected from six barley genotypes used for crossing with the Bob *AHAS* mutant are indicated by colored boxes (middle), where each color represents a unique allele and the white color represents the ‘Bob’-type allele. The total number of polymorphic markers identified per genotype pair with the Bob mutant is shown below. The PIC value calculated for each marker was plotted against its location on the genetic linkage map (right) to indicate the level of nucleotide diversity observed using 13 barley genotypes, and its distribution along the entire length of chromosome 6H.

Of the 61 markers used for analysis, two markers (*HvWaxy4* and *GBM1319*) were non-functional (no amplification observed in any of the genotypes), three markers (*HVM22*, *GBM1215* and *GMS6*) were monomorphic, and 56 markers were polymorphic. These polymorphic markers allowed us to detect 62 loci. Of the 56 polymorphic markers, one marker detected three loci, another marker detected two loci, while the remaining 52 markers each detected a single locus. (Fig. S2). These 56 markers amplified 1 to 12 alleles from the 13 barley genotypes (Table 2). The number of alleles detected by each marker and their frequencies were used to calculate the polymorphic information content (PIC) of the marker. The PIC value, which depends on the number of detectable alleles and the distribution of their frequency, indicates the marker’s utility in detecting polymorphism within a population [21]. The PIC values ranged from 0.14 (*84c21j33*, *GBM1355, GBM1212*) to 0.91 (*Bmac0144, Bmag0004*) (Table 2). When the PIC value for each marker was plotted against its location on the genetic-linkage map, it showed a multimodal distribution, with low levels of PIC values observed at the sub-telomeric and centromeric regions of the chromosome (Fig. 1). This distribution shows the level of nucleotide diversity along the entire length of the chromosome and suggests the possibility of identifying a polymorphic marker from a specific region of the chromosome. The type of repeat element, chromosomal location, number of repeat units, and sequence of repeat element can influence the level of nucleotide diversity. Thus, we classified the SSR markers according to the type of repeat element into simple and compound repeats. Whenever two or more repeat runs were present adjacent to each other or microsatellite array of same repeat was interrupted by non-repeat base(s) the repeat was classified as compound repeat. We further classified simple repeats into mono-, di-, tri-, tetra-, penta- and hexa-nucleotide repeats and reported their mean PIC values. Compound repeats in general showed higher PIC values in comparison with simple repeats, whereas, among simple repeats the di-nucleotide repeats showed highest PIC values (Table 3). To distinguish the effect of chromosomal location from the microsatellite element type, the PIC values obtained for different microsatellite types (i.e, mono-, di-, tri, tetra-, hexa-nucleotide repeats and compound repeats) were individually plotted against their respective location on the genetic-linkage map. The analysis revealed reduced levels of nucleotide diversity in the peri-centromeric region for di-nucleotide repeats and in sub-telomeric regions for the tri-nucleotide repeats (Fig. S3). However, it was apparent from the analysis that the number of repeat units does not have any influence on the number of alleles detected per locus.

**Table 3:** Microsatellite markers classified according to repeat element type.

Repeat type	SSR markers used	Mean PIC	Number of repeats
Simple	45	0.41	-
Mononucleotide	3	0.35	10
Dinucleotide	24	0.63	7 to 29
Trinucleotide	13	0.43	4 to 9
Trtranucleotide	3	0.33	5
Hexanucleotide	2	0.31	5
Compound	17	0.61	-

Number of simple sequence repeats (SSRs) or microsatellites falling in each category is listed and the range of alleles detected by SSRs in these categories and their average PIC (polymorphic information content) values are shown.

Preferential association of different SSR elements of variable sequences and lengths (i.e., total number of repeat units) with physical chromosome landmarks like the centromere, telomere, heterochromatin and euchromatin, and their relevance in determining chromosome function, has been extensively documented in literature [31]–[33]. Thus, the influence of the genomic locations of these markers on their evolvability and/or divergence is plausible. For instance, a low level of nucleotide diversity was observed in the proximal chromosomal regions of both *Triticum aestivum* and wild emmer (*Triticum turgidum* ssp. *dicoccoides*) [34]. Moreover, the effect of direct or indirect selection on genomic diversity is also a likely cause of observed fluctuations in genetic diversity along the chromosome length. Similar regions of low diversity associated with sites of domestication loci and genomic regions under selection in later breeding efforts were reported in maize [35]. Since barley genotypes selected in this study were bred in the PNW, they share some common ancestry. Thus, the regions of low diversity observed in the present study are likely to represent the genomic regions providing adaptive advantage to these genotypes. However, this aspect needs further investigation.

### Assessment of genetic diversity among barley genotypes

**Figure 2:**
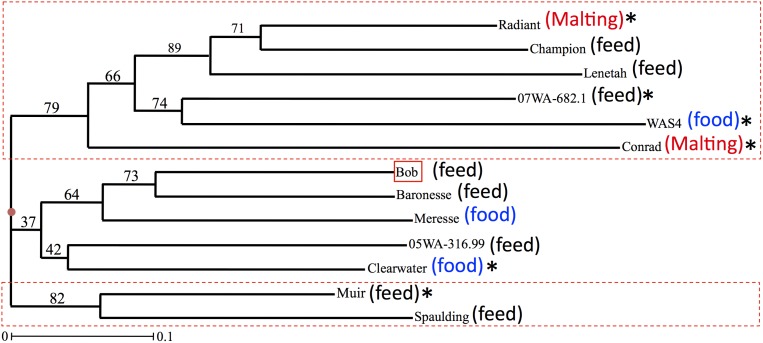
A dendrogram showing the clustering pattern of the 13 barley genotypes (see **Table 1** for genotype details). Genetic distances for the dendrogram were estimated using SSR (simple sequence repeat) polymorphism data. Bootstrap values are indicated at each node. The genotypes selected for crossing with the Bob *AHAS* mutant are marked with asterisk.

The genetic relationships among the barley genotypes were evaluated based on the combined profiles of 62 SSR loci. The genetic dissimilarity coefficient (GD) values were calculated for all possible 78 pairs of genotypes, and ranged from 0.339 (between Bob and Baronesse) to 0.806 (between WAS4 and Conrad) with a mean of 0.601 (Fig. S4). All 13 genotypes were grouped into three clusters (Fig. 2). Two clusters were further subdivided into two sub-clusters each. As expected on the basis of pedigree information (Table 1), Bob, Baronesse, Meresse, 05WA-316.99 and Clearwater formed a single cluster (middle), where the first three genotypes grouped into one sub-cluster and the latter two genotypes grouped into the other sub-cluster. Clustering of these genotypes in a single group can be explained by the presence of Baronesse in their lineages. The cultivars Radiant, Champion, Lenetah, 07WA-682.1, WAS4 and Conrad formed another cluster (top), where the first five genotypes formed a sub-cluster and Conrad alone formed a sub-cluster. The first sub-cluster was further divided into two sub-sub clusters, the former containing Radiant, Champion and Lenetah, and the later containing 07WA-682.1 and WAS4. The remaining two genotypes Spaulding and Lyon formed a separate cluster (bottom), which is well justified due to the Spaulding lineage of Lyon. The above diversity analysis proved useful in selecting lines to cross with the Bob *AHAS* mutant to transfer IMI-resistance, and will also prove useful in future breeding efforts where these lines will be used. Nevertheless, Baronesse has been extensively used in barley breeding programs in the PNW; the results clearly demonstrated high level of genetic diversity among studied genotypes, which is very important for the success of any breeding program. Thus, this study uniquely provides information about the genetic makeup of cultivars/breeding lines developed in the US PNW.

In summary, the polymorphism survey and diversity analysis i) allowed determination of genetic relationships of barley genotypes adapted to the US PNW; ii) provided data to make informed selection of barley genotypes used for crossing with the Bob *AHAS* mutant; iii) allowed identification of the most divergent pair of genotypes with the Bob mutant to be used for the genetic mapping of the *AHAS* gene; iv) allowed identification of the polymorphic markers for each pair of genotypes with Bob mutant to uniquely track and reconstitute the genetic-background of the recipient genotype; and v) allowed determination of the level of nucleotide diversity along the entire length of the barley chromosome 6H. This information not only proved useful during the present study but will also prove useful in later studies.

### Determination of the critical dose of herbicide

**Figure 3:**
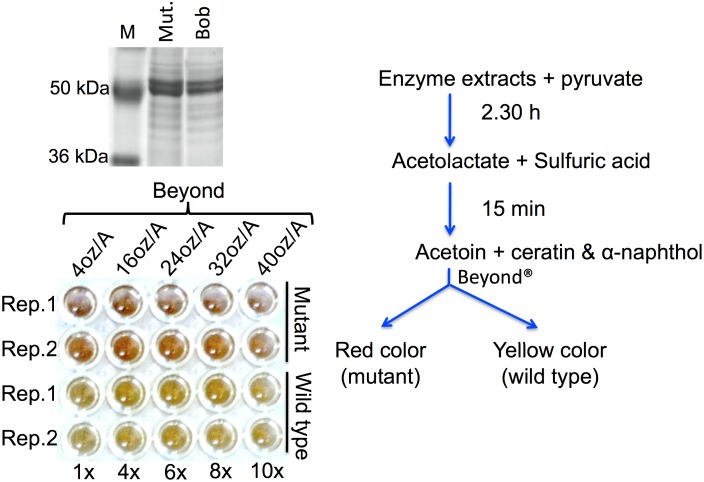
Results of the *in vitro* colorimetric enzyme activity assay performed for AHAS enzyme in the presence of inhibiting concentrations of an imidazolinone herbicide Beyond. Upper left, the crude enzyme was extracted from the leaves of ‘Bob’ and ‘Bob’ *AHAS* mutant and loaded on 10% SDS-polyacrylamide gel to check for the presence of the AHAS enzyme in the extracts. The presence of the enzyme in the extract was confirmed by a ∼65 kDa protein band on the gel. Lower left, the enzyme activity assay further confirms the presence of enzyme in the extracts from wild type and mutant. In the assay, the enzyme was fed with pyruvate (substrate) in presence of increasing concentrations of Beyond. The assay clearly showed that the mutant AHAS enzyme can survive up to 1.18 L/acre dose of herbicide and show equal activity if measured 15 min after addition of the color change solution (for more details, see Materials and Methods). The assay was performed as summarized in the line-diagram on the right.

From previous experience we know that the 0.118 L/acre dose of Beyond is sufficient to distinguish the susceptible barley genotypes from the resistant ones [16]. However, a critical herbicide dose, which could discriminate between the heterozygous and homozygous states of the *AHAS* mutation, remains unknown. Thus, in the present study, an attempt was made to determine the critical herbicide dose by spraying 0.118, 0.236 and 0.295 L/acre doses of Beyond on the segregating F_2_ population derived from WAS4×Bob mutant cross. A non-significant deviation from the 2∶1 segregation ratio (at p<0.05) of surviving vs dead plants was observed at each herbicide dose, which indicates the semi-dominant nature or dominant transmission of this mutation with incomplete penetrance (see next section for details). Subsequently, an effort was made to determine the maximum dose of herbicide, which can be tolerated by the IMI-resistant AHAS isoform. In order to achieve this objective, crude enzyme extracted from the leaf tissues of the Bob *AHAS* mutant was fed with the substrate (pyruvate) in presence of the increasing concentrations of the herbicide (see Materials and Methods). The assay suggested that the mutant enzyme can survive up to 1.18 L/acre Beyond that is 10 times field recommended dose applied on the IMI-tolerant winter wheat (Fig. 3). The assay also allowed discrimination of homozygotes from heterozygotes at 8× and 10× field recommended doses of the herbicide, displayed in the test by the intensity of red color as determined by the spectrophotometer. The heterozygotes took longer to produce same intensity of color that homozygotes produced in shorter duration of time (data not shown). However, these high doses of herbicide are impractical for use in glasshouse and field trials. In actual field conditions, the plant only receives a maximum of 0.236 L/acre dose, especially in the overlapping areas. Thus, for rest of the analyses, we used 0.236 L/acre herbicide dose.

Collectively these results suggested that the mutant AHAS enzyme can survive up to 10× field recommended dose of herbicide, which makes it unlikely to find a critical herbicide dose that can discriminate homozygotes from heterozygotes at the *AHAS* locus.

### Transfer of the IMI-resistance to other barley cultivars

A large collection of recombinants was screened in order to transfer IMI-resistance to selected genotypes in a single generation, and to identify rare recombinants carrying a small chromosomal segment with the gene of interest introgressed in the desired genetic background (Table 4). This will alleviate the need of backcrossing and avoid overriding the ‘Breeder’s Code of Ethics’. As mentioned in the Materials and Methods, the F_1_s were grown to obtain F_2_ seeds and a range of 2158 to 2846 F_2_ lines per cross combination were evaluated for the presence of the mutant allele. This has been achieved by spraying the F_2_ populations with 2× equivalent to the field recommended dose of Beyond used on the IMI-tolerant winter wheat (i.e., 0.236 L/Acre Beyond with 1% methylated seed oil), and by phenotyping the resistant plants for early vigor a month after spraying with the herbicide. The expected 3∶1 ratio of resistant vs susceptible plants, an indicative of the dominant nature of the mutation was not observed with any of the six segregating populations. Instead, the crosses between WAS4, Radiant, and Clearwater with the Bob mutant showed a 2∶1 segregation ratio of resistant vs susceptible plants (>0.05 probability). Collectively, the observed segregation ratios obtained from the greenhouse herbicide tests of the six segregating populations, at the best suggested a semi-dominant nature of the mutation or incomplete penetrance of the trait (Table 4). This low trait penetrance could be explained due to the cumulative effect of a number of factors like genetic differences for leaf and/or culm wax coating in the parental genotypes of a population, though this possibility needs further investigation.

**Table 4:** List of the number of crosses made, the F_1_ seeds obtained per cross combination and the F_2_ lines screened for imidazolinone (IMI)-resistance.

Female	Male	Crosses	F_1_ harvested	F_2_ sampled	F_2_ screened against herbicide	
					Susceptible	Resistant
**Feed**						
05WA-316.99	Bob mutant	53	471	2815	856	1959
07WA-682.1	Bob mutant	29	394	2251	813	1438
**Malting**						
Radiant*	Bob mutant	37	358	2130	669	1461
Conrad	Bob mutant	35	445	2671	806	1865
**Food**						
Clearwater*	Bob mutant	38	394	2336	790	1546
WAS4*	Bob mutant	38	403	2342	736	1606
**Total**		230	2465	14545	4670	9875

*Fitted in 2∶1 (resistant vs susceptible) segregation ratio at 0.05 significance level.

**Figure 4:**
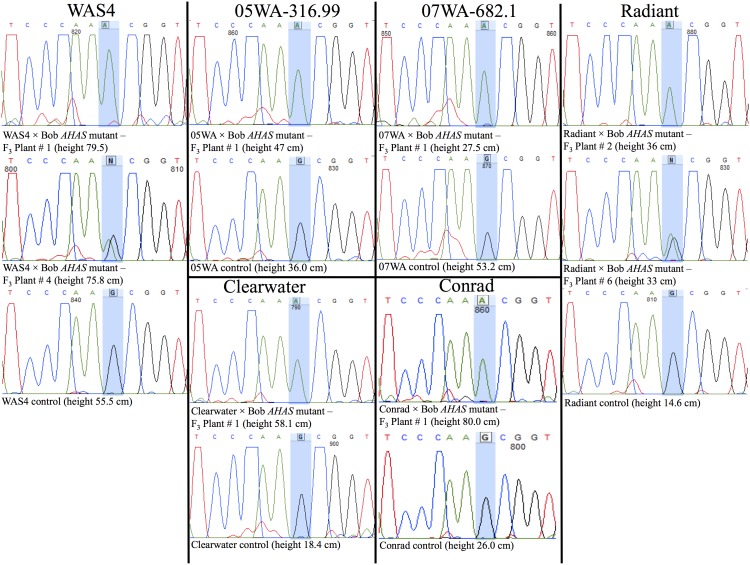
A part of the DNA sequence of the *AHAS* gene showing the point mutation responsible for IMI-resistance (highlighted in blue). The DNA sequencing results clearly demonstrated the transfer of IMI-resistance to two feed barleys, 05WA-316.99 and 07WA-682.1, two food barleys, Clearwater and WAS4, and two malting barleys, Radiant and Conrad.

**Figure 5:**
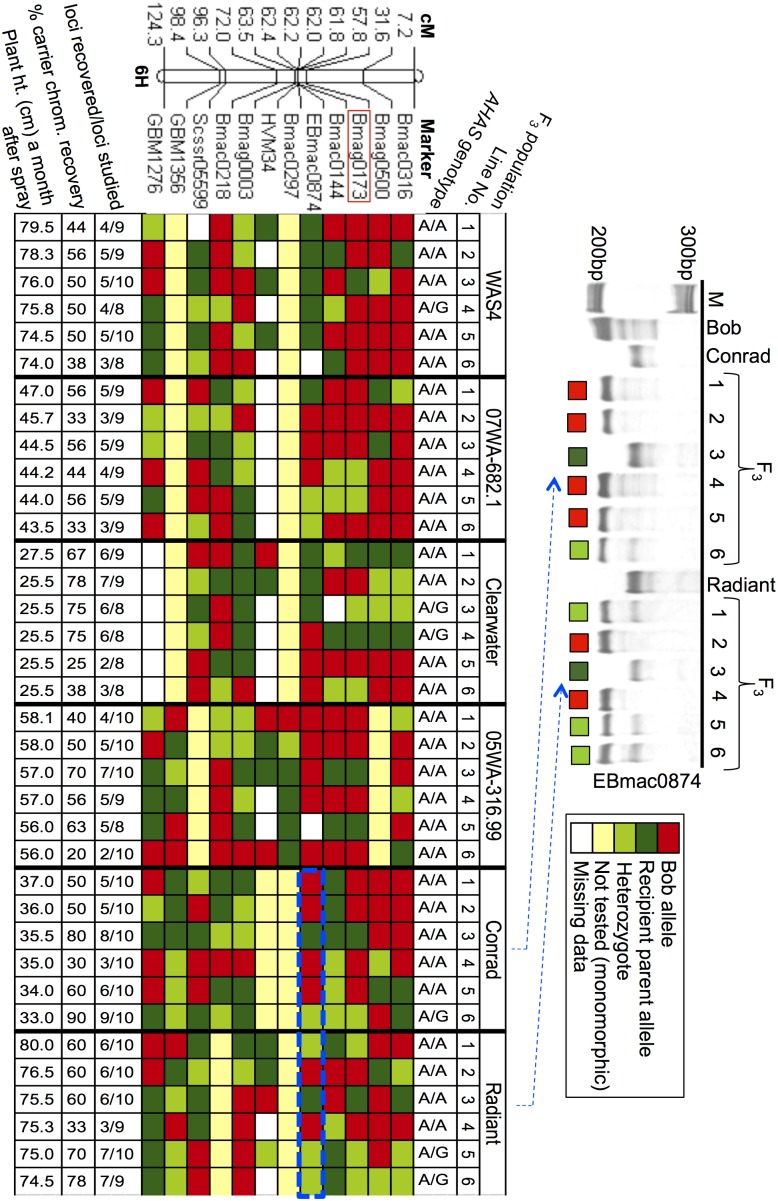
Diagrammatic representation of the results of the marker assisted background selection on the F_3_ progeny of the selected F_2_ lines. After foreground selection, the six F_3_ lines per cross combination were evaluated for the recovery of recipient parent background by genotyping each line with 10 to 12 chromosome 6H-specific microsatellite markers. The markers were selected on the basis of polymorphism data obtained earlier during diversity analysis and their respective location on chromosome 6H. Map locations of selected markers are shown on the left. Each column in the picture represents a F_3_ line and each row represents a DNA marker, whereas each cell represents the marker genotype in an individual. The marker genotype is represented by a color code: a) light green color denotes heterozygotes carrying marker alleles from both parents; b) dark green color denotes a marker allele similar to the recipient parent; and c) red color denotes the marker allele of the donor parent. Thus, a column with more dark and light green cells represent a genotype showing high percentage of the recipient parent genome, as observed for the 6^th^ F_3_ individual in the Conrad×Bob mutant cross, which had 90% recovery of the Conrad alleles. In contrast, a column showing more red cells represents a donor-like carrier chromosome as examplified by the 6^th^ F_3_ individual in the 05WA-316.99×Bob mutant cross, which had only 20% recovery of the 05WA-316.99 parent genome.

The semidominant nature of the mutant prompted us to determine the genotype at the *AHAS* locus (the foreground selection) by DNA sequencing of the *AHAS* gene fragment from 1 to 3 F_3_ lines each from the six most vigorous F_2_ plants selected per cross combination (Figs. 4 and 5). Although, an allele-specific agarose based assay exists for genotyping of segregating populations for the *AHAS* mutant allele, it is unsuitable for use in this situation due to its dominant nature (i.e., incapability of distinguishing between a heterozygote and a mutant type homozygote) [16]. Later, the six F_3_ plants showing the *AHAS* mutant allele in homo- or heterozygous state were selected to check for carrier chromosome recovery using 10–12 SSR markers specific to barley chromosome 6H. A range of 20 to 90% recovery of the recipient parent genome for the carrier chromosome was observed in the different cross combinations (Fig. 5). Collectively, this pilot study clearly demonstrates the feasibility of transferring IMI-resistance to desired barley genotypes in a single generation with the possibility of finding lines showing good recovery of the recipient parent genome.

## Conclusion

Results of the study are of high significance not only to growers in the Pacific Northwest but also to growers in other parts of the US and the world, wherever IMI-herbicides are applied and IMI-resistant crops are cultivated. In this study we determined the genetic diversity among 13 barley cultivars/breeding lines, which benefitted the present study and is expected to prove useful in future breeding efforts. Chromosomal localization of the gene encoding the catalytic subunit of the barley AHAS enzyme will also prove useful in future gene-transfer studies leading to the development of herbicide-resistant cultivars with other agronomically important traits. Determination of the working dose of herbicide used for phenotypic screening of this trait will be used in future breeding efforts to transfer IMI-resistance. This pilot study with a limited number of selected F_2_ lines shows that it is possible to identify genotypes showing good recovery of the recipient parent genome by screening large F_2∶3_ populations and following a strategic selection scheme (Fig. 5).

Our future objective is to take the recently developed IMI-resistant food, feed and malting barley genotypes from the glasshouse to the field by i) screening large numbers of F_3_ families, representing the 250 top ranking F_2_ lines selected per cross combination, based on their vigor a month after herbicide spray, for their genetic backgrounds using DNA markers; ii) fixing heterozygosity (which confounds phenotypic evaluations) in selected lines by doubled haploid (DH) production; iii) field evaluation of the DH lines for their performance on herbicide residue and under spray trials. This will allow identification of barley lines showing more genetic proximity to their respective recipient parents.

For the first objective, F_3_ seeds belonging to the 250 F_2_ lines which survived the herbicide spray (at the rate of 0.236 L/acre Beyond with methylated seed oil) and showed early vigor a month after spray are currently being propagated in herbicide treated soil in the glasshouse. Cultivating plants on herbicide treated soil will allow elimination of susceptible individuals, which are expected in a segregating population at a proportion of one in four individuals. Genotype of the survivors will be determined at the AHAS locus by DNA sequencing following the procedure described above. It is of considerable importance to differentiate homozygotes from heterozygotes at the AHAS locus, as the two genotypic states at this locus are undistinguishable from each other using herbicide treatment alone. This is due to the semi-dominant nature of the AHAS mutation. The lines possessing the mutant allele(s) at the AHAS locus either in homo- or heterozygous state will be evaluated for their genetic background in a stepwise fashion first using 10 carrier chromosome (6H) specific microsatellite markers followed by 4 DNA markers per non-carrier chromosomes (2 markers per arm). The second step of background selection will be performed on the F_3_ plants showing good carrier chromosome recovery in the first step. The lines showing good recovery of recipient parent genome will be converted to doubled haploids via a microspore culture based method following Kasha et al. [36]. The resultant doubled haploids will be evaluated for their performance in the field on herbicide residue and herbicide spray trials.

The major outcome of this project will be the development of IMI-resistant barley varieties and germplasm with a combination of beneficial traits including resistance for various biotic and abiotic stresses, higher grain yield and better quality. Moreover, adding imidazolinone resistance to barley cultivars adapted to the PNW will certainly improve the sustainability of barley, which is one of the best rotational crops for this region.

## Supporting Information

Figure S1 **Comparative mapping of wheat chromosome 6D and barley chromosome 6H to determine approximate location of the** ***AHAS*** **gene on chromosome 6H.** (a) Genetic linkage map of wheat chromosome 6D. (b) Physical map of wheat chromosome 6D. Short arm is at the top, and the black circle indicates the centromere. Deletion-line breakpoints and fraction lengths (FLs) are indicated by the horizontal line to the left. Breakpoint positions are drawn approximately to scale. Darkened areas within chromosome arms are C-bands (cf. Endo and Gill. 1996. Journal of Heredity 87∶295). (c) Microsatellite consensus map of barley chromosome 6H (modified from Varshney et al. 2007. Theoretical and Applied Genetics 114∶1091). (d) Genetic location of the *AHAS* gene determined on the basis of *in silico* analysis. The gene was assigned to the ‘Morex’ BAC-contig #40275 anchored to the consensus genetic linkage map at 67.917 cM (cf. Close et al. 2009. BMC Genomics 10∶582).
(TIFF)

Figure S2 **Consensus map of barley chromosome 6H (left; Varshney et al. 2007 Theoretical and Applied Genetics 114∶1091) used to select simple sequence repeat (SSR)-markers for diversity analysis of two-rowed spring barley genotypes.** Amplification profile of a few SSR markers used for analysis of barley genotypes are shown on left, and their locations on the genetic-linkage map are highlighted by red rectangles. Different SSR alleles are coded by different numbers and shown on the bottom of each SSR profile.
(TIFF)

Figure S3 **Polymorphic information content (PIC) values for different SSRs (classified according to repeat element type) are plotted against their respective location (in cM) on the genetic linkage map, showing variation in nucleotide diversity observed along the entire length of chromosome 6H.**
(TIFF)

Figure S4 **The dissimilarity coefficient (GD) values calculated for 78 pairs of genotypes.** High to low dissimilarity coefficient values with ‘Bob’ are shown on a red to white scale, with the highest value (0.726) shaded with the darkest red color, and the lowest value (0.435) in white.
(TIFF)
