# Supplementary figures and images for: Assessment of Genetic Diversity among Barley Cultivars and Breeding Lines Adapted to the US Pacific Northwest, and Its Implications in Breeding Barley for Imidazolinone-Resistance
Source: PLoS One. 2014 Jun 26;9(6):e100998. doi: 10.1371/journal.pone.0100998 (PMC4072767; doi:10.1371/journal.pone.0100998)

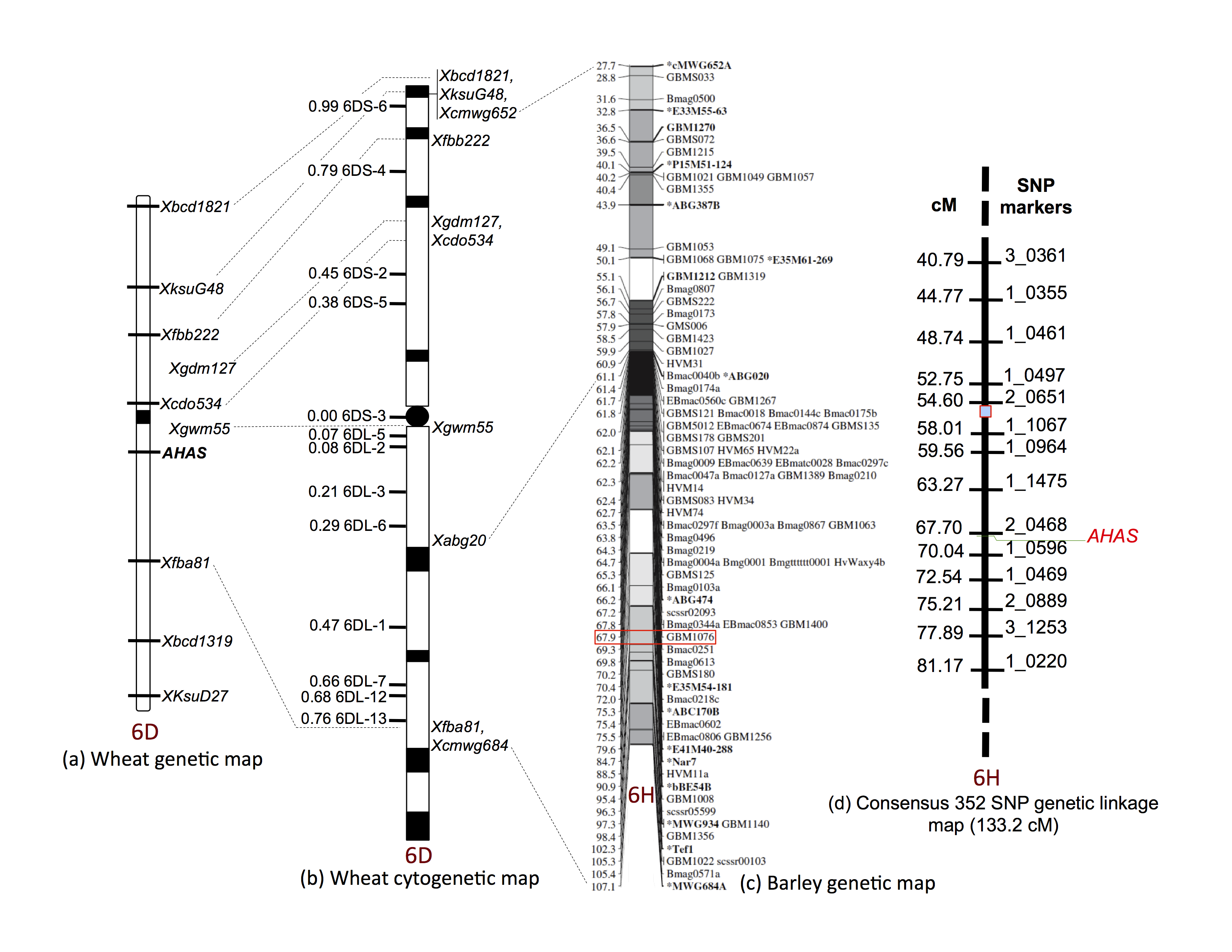

Supplement: Figure S1 — Comparative mapping of wheat chromosome 6D and barley chromosome 6H to determine approximate location of the AHAS gene on chromosome 6H. (a) Genetic linkage map of wheat chromosome 6D. (b) Physical map of wheat chromosome 6D. Short arm is at the top, and the black circle indicates the centromere. Deletion-line breakpoints and fraction lengths (FLs) are indicated by the horizontal line to the left. Breakpoint positions are drawn approximately to scale. Darkened areas within chromosome arms are C-bands (cf. Endo and Gill. 1996. Journal of Heredity 87∶295). (c) Microsatellite consensus map of barley chromosome 6H (modified from Varshney et al. 2007. Theoretical and Applied Genetics 114∶1091). (d) Genetic location of the AHAS gene determined on the basis of in silico analysis. The gene was assigned to the ‘Morex’ BAC-contig #40275 anchored to the consensus genetic linkage map at 67.917 cM (cf. Close et al. 2009. BMC Genomics 10∶582). (TIFF) [file pone.0100998.s001.tiff]

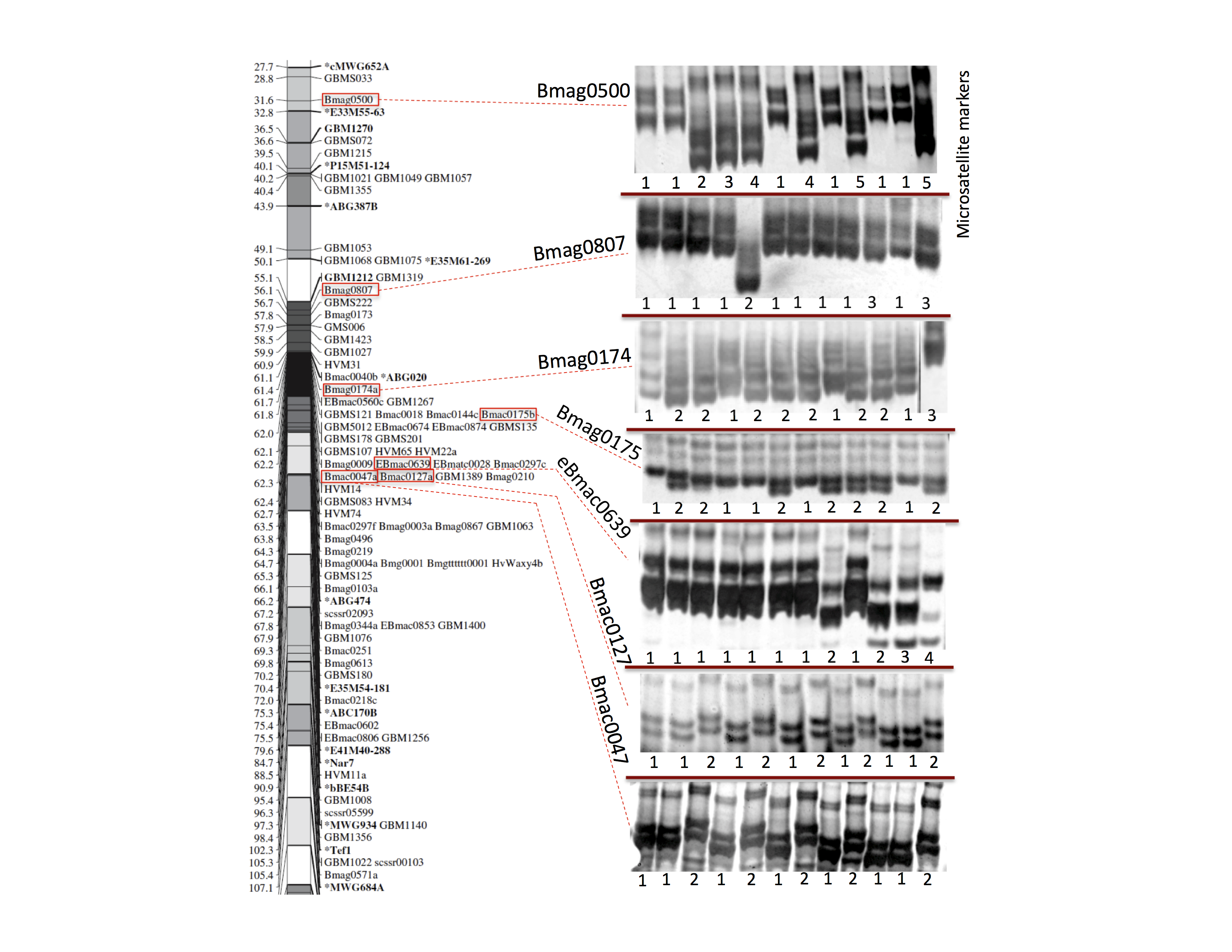

Supplement: Figure S2 — Consensus map of barley chromosome 6H (left; Varshney et al. 2007 Theoretical and Applied Genetics 114∶1091) used to select simple sequence repeat (SSR)-markers for diversity analysis of two-rowed spring barley genotypes. Amplification profile of a few SSR markers used for analysis of barley genotypes are shown on left, and their locations on the genetic-linkage map are highlighted by red rectangles. Different SSR alleles are coded by different numbers and shown on the bottom of each SSR profile. (TIFF) [file pone.0100998.s002.tiff]

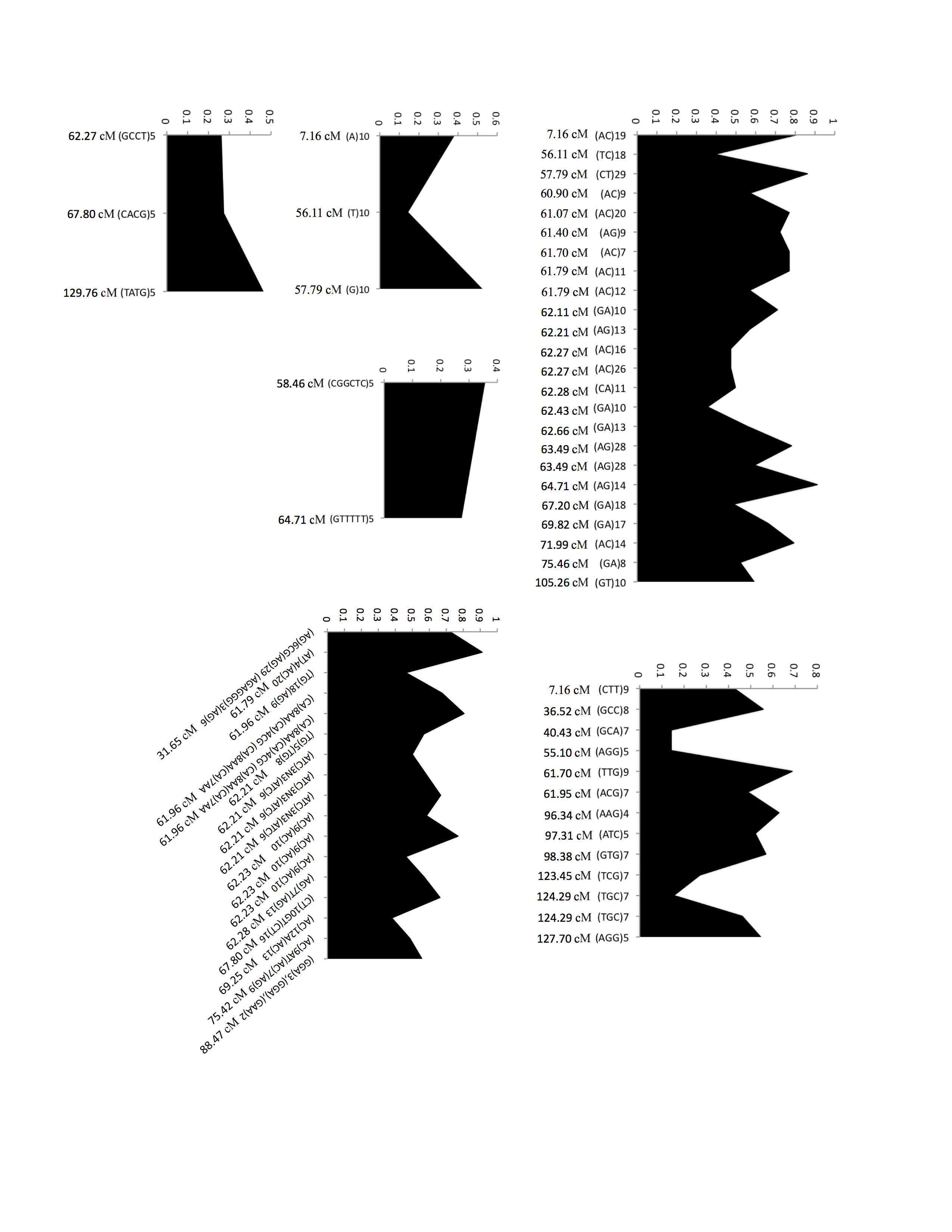

Supplement: Figure S3 — Polymorphic information content (PIC) values for different SSRs (classified according to repeat element type) are plotted against their respective location (in cM) on the genetic linkage map, showing variation in nucleotide diversity observed along the entire length of chromosome 6H. (TIFF) [file pone.0100998.s003.tiff]

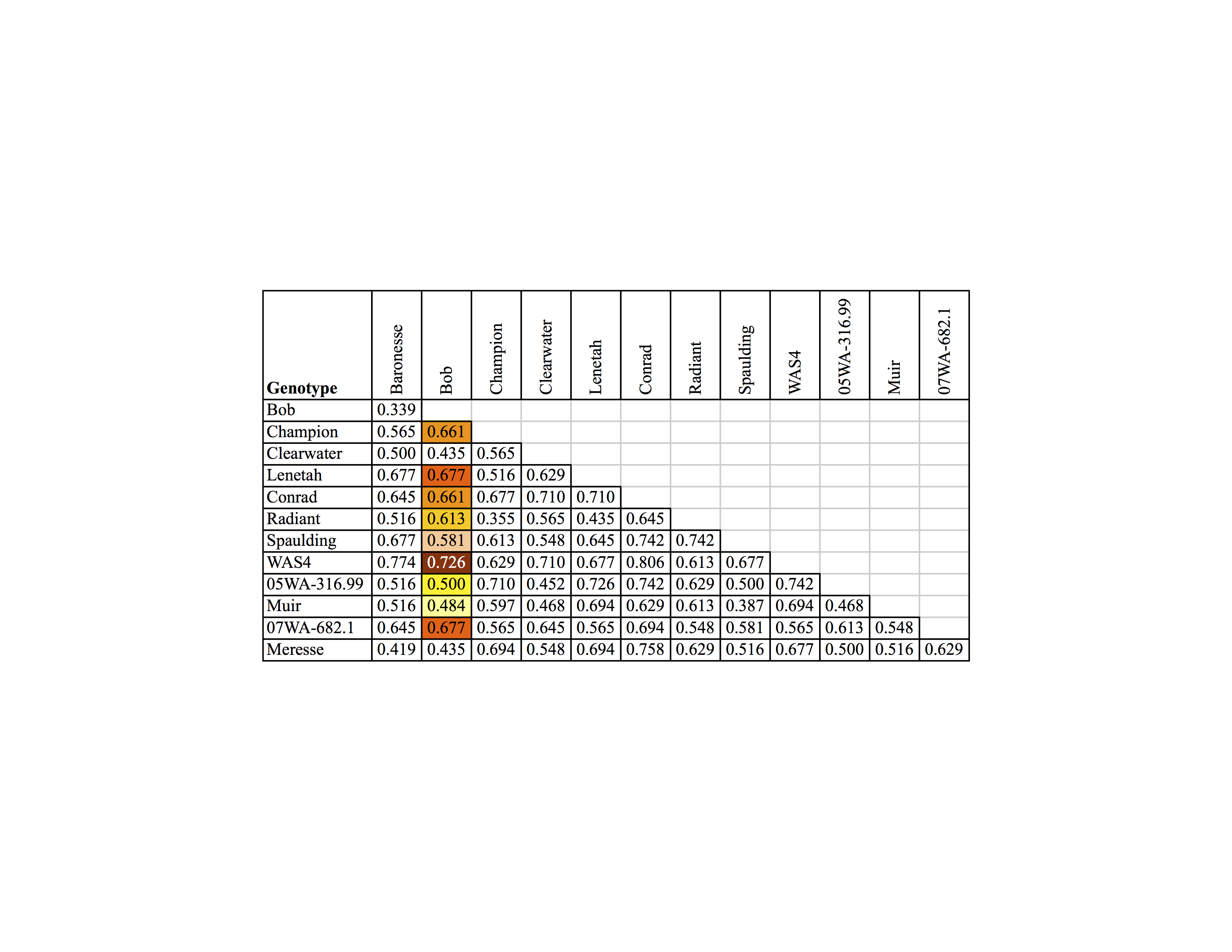

Supplement: Figure S4 — The dissimilarity coefficient (GD) values calculated for 78 pairs of genotypes. High to low dissimilarity coefficient values with ‘Bob’ are shown on a red to white scale, with the highest value (0.726) shaded with the darkest red color, and the lowest value (0.435) in white. (TIFF) [file pone.0100998.s004.tiff]
